# Role of synergy and immunostimulation in design of chemotherapy combinations: An analysis of doxorubicin and camptothecin

**DOI:** 10.1002/btm2.10129

**Published:** 2019-06-13

**Authors:** Anusha Pusuluri, Vinu Krishnan, Debra Wu, C. Wyatt Shields, Li W. Wang, Samir Mitragotri

**Affiliations:** ^1^ John A. Paulson School of Engineering and Applied Sciences, Harvard University Cambridge MA 02138; ^2^ Wyss Institute of Biologically Inspired Engineering, Harvard University Boston MA 02115; ^3^ Department of Chemical Engineering University of California Santa Barbara CA 93106; ^4^ Harvard–MIT Health Sciences and Technology Program Institute for Medical Engineering and Science, Massachusetts Institute of Technology Cambridge MA 02139

**Keywords:** camptothecin, doxorubicin, immune activation, ratiometric drug dosing, synergy, triple negative breast cancer

## Abstract

Combination chemotherapy is often employed to improve therapeutic efficacies of drugs. However, traditional combination regimens often utilize drugs at or near‐their maximum tolerated doses (MTDs), elevating the risk of dose‐related toxicity and impeding their clinical success. Further, high doses of adjuvant or neoadjuvant chemotherapies can cause myeloablation, which compromises the immune response and hinders the efficacy of chemotherapy as well as accompanying treatments such as immunotherapy. Clinical outcomes can be improved if chemotherapy combinations are designed to reduce the overall doses without compromising their therapeutic efficacy. To this end, we investigated a combination of camptothecin (CPT) with doxorubicin (DOX) as a low‐dose treatment option for breast cancer. DOX‐CPT combinations were synergistic in several breast cancer cell lines in vitro and one particular ratio displayed extremely high synergy on human triple negative breast cancer cells (MDA‐MB‐231). This combination led to excellent long‐term survival of mice bearing MDA‐MB‐231 tumors at doses roughly five‐fold lower than the reported MTD values of its constituent drugs. Impact of low dose DOX‐CPT treatment on local tumor immune environment was assessed in immunocompetent mice bearing breast cancer (4T1) tumors. The combination was not only superior in inhibiting the disease progression compared to individual drugs, but it also generated a more favorable antitumor immunogenic response. Engineering DOX and CPT ratios to manifest synergy enables treatment at doses much lower than their MTDs, which could ultimately facilitate their translation into the clinic as a promising combination for breast cancer treatment.

## 1 INTRODUCTION

Combination chemotherapy, in spite of its limitations, is the current gold standard for the treatment of advanced breast cancers.^1^, ^2^ A meta‐analysis of several combination therapies for the treatment of metastatic breast cancer revealed that a heterogeneous yet statistically significant benefit was obtained for combination regimens in terms of tumor progression and overall survival.^3^ Notwithstanding these improvements, the median survival times in combination treatment are still low and the survival benefits are counterbalanced with a proportional increase in the toxicity contributing to severe morbidity and poor quality of life for patients. This is because combination chemotherapies typically employ delivery of their components at their maximum tolerated doses (MTDs) under the assumption that they have nonoverlapping toxicities.^4^, ^5^ However, in the clinic, patients are simultaneously exposed to near toxic doses of multiple poorly tolerable agents that manifest increased adverse effects and eventually undermine the intended therapeutic benefit. Further, dosing chemotherapeutic drugs at their MTDs typically causes dose‐limiting toxicities such as myelosuppression and febrile neutropenia, which makes the tumors notoriously immunosuppressive,^6^ even if they are treated with drugs known to have strong anticancer immunogenic effects.^7^, ^8^ While the modest observed benefits support the efforts to employ combinations in the clinic; they also highlight the urgent need for identifying additional ways to improve the treatment outcomes and design more effective therapies. Emerging studies show that different molar ratios of a given drug combination can have different cell‐killing effects and several synergistic drug pairs have been empirically identified and tested both in vitro and in vivo.^9^, ^10^, ^11^, ^12^, ^13^, ^14^, ^15^, ^16^ Although there is a growing consensus on combining chemotherapy drugs at specific molar ratios to afford higher potency and yield effective responses at greatly diminished drug doses, their impact on the intratumoral immune response is rarely assessed.

In this study, we propose one such low dose therapy using two topoisomerase inhibitors, doxorubicin (DOX) and camptothecin (CPT), for the effective management of an aggressive triple negative breast cancer and also study its effect on the intratumoral immune microenvironment. Topoisomerase I inhibitors have gained widespread attention as well tolerated drugs in managing refractory metastatic breast cancers after treatment with anthracyclines and taxanes, the most widely indicated drug classes in breast cancer.^17^, ^18^ Clinically approved topoisomerase I inhibitors like irinotecan and topotecan have experienced limited success due to the provision of minimal therapeutic benefit coupled with significant worsening in toxicity.^19^, ^20^ Nevertheless, the synergistic interactions of CPT, an extremely potent topoisomerase I inhibitor,^21^ with topoisomerase II inhibitors like DOX, which have been described in several in vitro and in vivo studies warranted an evaluation of this drug combination.^14^, ^22^, ^23^ CPT has not been approved in the clinic so far due to its unpredictable toxicity in patients at high doses coupled with variable and limited objective responses in phase II clinical trials.^21^ We hypothesized that by leveraging the synergistic interactions between DOX and CPT and optimally combining them, effective therapeutic responses may be achieved at significantly reduced doses that can eventually be well tolerated in the clinic.

We systematically evaluated combinations of DOX and CPT at different ratios in several breast cancer cell lines, and found that the efficacy of the combination depends strongly on the drug ratio. The optimized ratio induced a substantial reduction in tumor burden at low and well‐tolerated drug doses (2 mg/kg/dose of DOX and 1.2 mg/kg/dose of CPT) in an aggressive in vivo triple negative breast cancer model. We also show that, when combined, the drug doses used in this study can elicit an effective antitumor immune effect in a syngeneic breast cancer model.

## 2 MATERIALS AND METHODS

### 2.1 Materials

MDA‐MB‐231, 4T1 and MCF7 cells were obtained from ATCC (Manassas, VA). RPMI‐1640 media, DMEM media, fetal bovine serum (FBS), penicillin and streptomycin (Pen Strep), 3‐(4,5‐dimethylthiazol‐2‐yl)‐2,5‐diphenyltetrazolium bromide (MTT), heparin‐coated plasma preparation tubes, Gibco™ Type 1 Collagenase, ACK lysing buffer, Invitrogen™ UltraComp eBeads™ Compensation Beads and SYTOX™ Blue dead cell stain were purchased from Thermo Fisher Scientific (Waltham, MA). Cell culture flasks, microplates and matrigel were purchased from Corning (Corning, NY). Doxorubicin (DOX) was purchased from LC Laboratories (Woburn, MA) and DNAase I was purchased from Roche (Indianapolis, IN). Cell strainers (70 μm) were purchased from BD Biosciences (San Jose, CA); rat and mouse serum were purchased from Bio‐Rad (Hercules, CA). Anti‐mouse CD16/32 antibody was purchased from BioLegend (San Diego, CA). Camptothecin (CPT), Tween‐80 and all other chemicals were purchased from Sigma–Aldrich (St. Louis, MO). Information about the antibodies and their corresponding clones and fluorophores are detailed in the supplementary information (Table S1).

### 2.2 Cell culture

MDA‐MB‐231 and 4T1 cells were cultured in RPMI‐1640 medium supplemented with 10% FBS and 1% Pen Strep and maintained in a humidified incubator with 5% CO_2_ at 37°C. MCF7 cells were cultured similarly in DMEM medium supplemented with 10% FBS and 1% Pen Strep. Flasks were subcultured when the cells were approximately 80% confluent.

### 2.3 In vitro cell toxicity assay and synergy analysis

A suspension of 2.5 × 10^3^ 4T1 cells or 5 × 10^3^ MDA‐MB‐231 or 5 × 10^3^ MCF7 cells in 100 μL media were seeded per well and allowed to adhere overnight in a 96 well culture plate. The media was aspirated and exchanged for serial dilutions of drug cocktails, containing DOX and/or CPT at the desired molar ratio, prepared in fresh media. Drugs were incubated for 72 hr after which cell viability was assessed using the MTT assay. A volume of 100 μL MTT solubilized in media at 0.5 mg/mL was added and allowed to incubate for 3.5 hr after aspirating the drug solutions. Finally, the MTT solution was aspirated and replaced with DMSO and the well plates were left on a shaker for 20 min at 350 rpm to solubilize the formazan crystals. Absorbance from each well at 570 nm was read (Tecan Infinite M1000) and cell viability (fraction affected, *f*_*a*_) was calculated as follows:

$$f_{a}=1-\frac{A_{i}-A_{\text{Blank}}}{A_{0}-A_{\text{Blank}}},$$

where *A*_*i*_, *A*_Blank_ and *A*_0_ are absorbance values from a treatment well *i*, a blank well and a control well, respectively. To generate in vitro cytotoxicity curves and the half maximal inhibitory concentration (IC_50_) values, experimental cell viability data were fitted to the median‐effect model.^24^ Synergy was then assessed and optimal ratios for DOX and CPT were identified using the previously described Chou‐Talalay Combination Index (CI) method.^10^, ^13^, ^14^ Synergism, additivism and antagonism are indicated by CI values less than 1, equal to 1 and greater than 1, respectively. The following formula was used to calculate CI:

$$\mathrm{CI}=\frac{{\left(\text{Comb}\;{\mathrm{IC}}_{50}\right)}_{\mathrm{DOX}}}{{\left({\mathrm{IC}}_{50}\right)}_{\mathrm{DOX}}}+\frac{{\left(\text{Comb}\;{\mathrm{IC}}_{50}\right)}_{\mathrm{CPT}}}{{\left({\mathrm{IC}}_{50}\right)}_{\mathrm{CPT}}},$$

where (Comb IC_50_)_DOX_ and (Comb IC_50_)_CPT_ are the IC_50_ values in a combination and (IC_50_)_DOX_ and (IC_50_)_CPT_ are individual IC_50_ values of DOX and CPT, respectively. CI errors are reported by propagating the *SE*s in the IC_50_ values from the corresponding drug model fits.

### 2.4 In vivo tumor growth inhibition

Experiments pertaining to the use of animals were performed according to the protocols approved by the Institutional Animal Care and Use Committee of Harvard University and University of California Santa Barbara. Orthotopic xenografts of the breast cancer cell line MDA‐MB‐231 were developed by injecting 2.5 × 10^6^ cells (>98% cell viability) in 100 μL of 1:1 matrigel and saline into the subcutaneous space of the lower left inguinal mammary fat pad in athymic nu/nu mice aged between 42 and 56 days using a 25 G needle. Similarly, a 4T1 orthotopic mouse breast cancer model was developed by injecting 1 × 10^5^ cells (>98% cell viability) in 50 μL of saline subcutaneously into the lower left inguinal mammary fat pad of balb/c mice between 42 and 56 days in age.

Tumor bearing mice were randomized before treatments and monitored for tumor growth and body weight changes throughout the study. Mice received a total of four treatments, which began 11 days post inoculation in the MDA‐MB‐231 model and 9 days post inoculation in the 4T1 model (tumor volume in both cases ~ 50 mm^3^). Drug formulations were dissolved in sterile saline (0.9 wt/vol% NaCl), containing 10 vol% of Tween‐80, and were administered every other day via tail vein injections. Tumor volumes (V) and tumor growth inhibition (TGI) were calculated using the following formulae:

$$V=\frac{1}{2}\;l\times w^{2},$$

$$\mathrm{TGI}\;\left(\%\right)=100*\left(1-\frac{V_{\text{treatment}}\left(\text{final}\right)-V_{\text{treatment}}\left(\text{initial}\right)\;}{V_{\text{control}}\left(\text{final}\right)-V_{\text{control}}\left(\text{initial}\right)}\;\right),$$

where *l* and *w* are the longest and shortest dimensions of the tumor, *V*_treatment_ and *V*_control_ are tumor volumes of mice receiving drug treatments or PBS and *initial* and *final* represent the first and last day of the study, respectively. Mice were euthanized if *l* exceeded 15 mm, or if body weight loss exceeded 15%, or if necrotic ulcers became visible.

### 2.5 Phenotyping tumor‐associated immune‐cell population

4T1 tumors were harvested 8 days after administering the last treatment, snipped into small pieces (<5 mm in thickness) and enzymatically degraded at 37°C for 90 min in HBSS buffer containing 5 mg/mL collagenase type I, 50 U/mL of DNAse I and 5% FBS. To form single cell suspensions, the enzyme‐tumor mixture, diluted in PBS containing 50 U/mL of DNAse I, was passed through 70 μm cell strainers with the aid of gentle trituration as needed. Cells were then centrifuged and resuspended in ACK red cell lysis buffer supplemented with 50 U/mL of DNAse I for 5 min. Cells were again centrifuged and resuspended in PBS to obtain the total cell count of the remaining intact cells. For the remainder of this study, 1 × 10^6^ live cells per tumor were used and all steps were performed in 100 μL FACS buffer (PBS with 3% FBS and 30 μM EDTA) supplemented with additional reagents as necessary. Cells were first blocked for 30 min in a solution consisting of 5% rat serum, 5% mouse serum and 1% anti‐mouse CD16/32 antibody. Next, cells were stained with control and test antibodies (according to the gating strategy shown in Scheme S1) for 30 min at room temperature and for 20 min on ice in a dark enclosed space. Cells were then washed twice with ice‐cold FACS buffer and resuspended in 500 μL of PBS. Stained cells were analyzed for surface markers via flow cytometry (BD LSRII). Cells stained with SYTOX™ Blue dead cell stain as per the manufacturer's protocol were used to measure cell viability at the end of all treatment steps. All compensation and voltage settings were determined by using compensation beads stained with one antibody at a time and data obtained were analyzed using FCS Express 6 software (De Novo Software, Glendale, CA). All centrifugation steps were done at 250 × *g* for 5 min.

### 2.6 Statistical analysis

All analyses and comparisons were performed using GraphPad Prism 6. Except for survival data, significant differences between groups in all other data were determined by performing, multiple *t* tests, one‐way ANOVA or two‐way ANOVA, as applicable, and adjusted for multiple comparisons by the Tukey–Kramer method (*α* = 5%). Mantel‐Cox test was used for comparing and determining significant differences between two groups in survival curves.

## 3 RESULTS

### 3.1 Ratio‐dependent synergy between DOX and CPT

Three different breast cancer cell lines MDA‐MB‐231, MCF 7 and 4T1, each with different relative sensitivities to DOX and CPT, were used to assess the ratio‐dependent synergy between DOX and CPT. MDA‐MB‐231 cells were more sensitive to CPT (DOX‐IC_50_ > CPT‐IC_50_), MCF 7 cells displayed similar sensitivity to both DOX and CPT (DOX‐IC_50_ ~ CPT‐IC_50_) and 4T1 cells were more sensitive to DOX (DOX‐IC_50_ < CPT‐IC_50_) (Figure S1). Molar ratios were chosen for testing based on these relative efficacies such that, at any given ratio, both drugs are expected to contribute to the overall cytotoxicity without overwhelming the other drug's effect. Synergy was quantified by calculating the combination index (CI) using drug doses obtained by median effect analysis, as described previously.^10^ Using this method, drug interactions at different ratios can be evaluated at different effect levels (fraction of cells affected) and CI can be expressed for any effect level. However, since the median effect represents a linear approximation of a nonlinear function, the plot may be unreliable at the extremes. Hence, the most accurate CI determination is at the dose where 50% of cell growth is inhibited or in other words at the IC_50_ value for each drug.^2^

**Figure 1:**
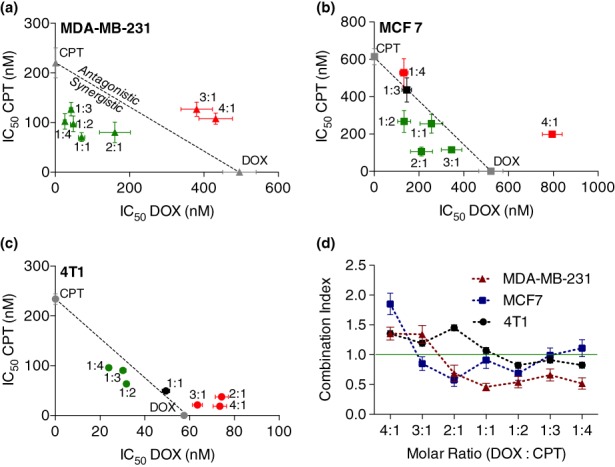
Effect on (a) MDA‐MB‐231, (b) MCF 7, and (c) 4T1 cell proliferation due to variation in the molar ratio of DOX and CPT. IC_50_ data of each DOX:CPT ratio are expressed on the isobolograms as mean ± *SE* obtained from the corresponding drug model fits (*n* ≥ 5). The dotted diagonal line is the locus of points representing the doses, at all possible ratios of DOX and CPT, that are additive. All points below the line and towards the axes are synergistic (green); all points on the line are additive (black); and all points above the line and away from the axes are antagonistic (red). (d) in vitro assessment to quantify DOX and CPT synergy by Chou‐Talalay combination index (CI) method for identifying optimal drug molar ratios. The green line represents CI = 1 (additive effect), CI < 1 is synergistic and CI > 1 is antagonistic. Error in CI was calculated by propagating *SE*s in IC_50_ values from the corresponding ratio (*n* ≥ 5). Statistical analysis was performed by two‐way ANOVA and all significance information is provided in the supplementary information (Table S2). CPT, camptothecin; DOX, doxorubicin

Overall, synergy was most commonly seen in MDA‐MB‐231 cells, where 5 of the 7 combinations displayed strong synergy (Figure 1a, S2A). The highest synergy in MDA‐MB‐231 cells was achieved at a ratio of 1:1 DOX:CPT (CI = 0.46 ± 0.06). At this ratio, the dose reduction observed was a remarkable 86% (seven‐fold) for DOX and 68% (three‐fold) for CPT. The next best synergy was seen in MCF 7 cells (Figure 1b, S2B) where, 4 of the 7 ratios tested were synergistic, 1 was additive and 2 were antagonistic. Here, the 2:1 ratio of DOX:CPT presented the best synergy (CI = 0.58 ± 0.11). Finally, DOX and CPT were least synergistic in inhibiting the 4T1 cell growth (Figure 1c, S2C). Here, 3 of the 7 ratios were weakly synergistic (the best synergy achieved at 1:2 DOX:CPT, CI = 0.82 ± 0.05) and of the remaining ratios, 1 was additive and 3 were antagonistic.

Interestingly, irrespective of the relative sensitivity, ratios with higher amounts of CPT in the drug cocktail resulted in higher synergies for all cell lines (i.e., CI < 1.0; Figure 1d). While in most cases the individual contribution of each drug (IC_50_ of DOX/CPT) towards the cumulative cell‐killing diminished with decreasing relative concentration in the cocktail (Figure S2), there were some cases where such monotonic dependence was not observed. For example, in MDA‐MB‐231 cells, the IC_50_ of CPT varied across the different ratios tested and did not follow any particular trend (Figure S2A). In MCF 7 cells, with increasing levels of CPT in the drug cocktail, the IC_50_ of CPT initially decreased until the 2:1 ratio of DOX:CPT and later increased with increasing relative concentration of CPT in the drug cocktail (Figure S2B).

### 3.2 Low dose in vivo tumor growth inhibition by DOX and CPT in an MDA‐MB‐231 murine model

Based on the high in vitro synergy between DOX and CPT in MDA‐MB‐231 cells in vitro, we pursued this drug pair for further in vivo studies. The highest synergy was observed at a molar ratio of 1:1 (DOX:CPT), which corresponded to a combination treatment that achieved similar efficacies at significantly reduced doses compared to their single drug counterparts. To examine if this would translate into needing considerably lower drug doses for effective tumor reductions in vivo, we performed a dose escalation study with the drug cocktail at the same ratio. Doses were chosen to be well below the MTD of DOX (between 8 and 12 mg/kg/dose)^25^, ^26^ and CPT (15 mg/kg/dose).^27^ Since higher CPT doses would likely increase the risk of toxicity, we chose doses such that the final CPT dose was at least 10–100 fold lower than its MTD.

**Figure 2:**
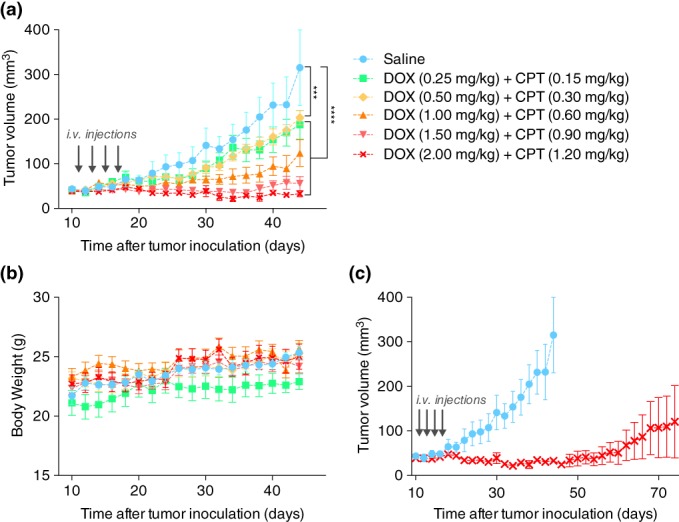
In vivo efficacy of DOX and CPT combination treatments at different dose levels in an orthotopic MDA‐MB‐231 mouse breast cancer model. (a) Tumor growth curves for different treatment doses of DOX and CPT at a 1:1 M ratio. Starting on Day 11 postinoculation, four i.v. injections (gray arrows) were administered every other day. Statistically significant differences, obtained by performing one‐way ANOVA, are shown for the last day on the growth curve (Day 44; ***p < .001 and ****p < .0001). (b) Corresponding body weight changes for all treatment groups. (c) Long term tumor growth data for groups treated with 2 mg/kg DOX and 1.2 mg/kg CPT or saline. Data for either group were plotted until the day when one or more mice were sacrificed as per the euthanasia criteria listed in the methods section. Corresponding body weight changes and survival curves are provided in Figure S3. All data are expressed as mean ± SEM (*n* = 5). CPT, camptothecin; DOX, doxorubicin

Four injections of either saline or of different low dose cocktails of DOX + CPT were administered intravenously (i.v.) every other day (q2d × 4) in athymic nude mice with MDA‐MB‐231 tumors in the mammary fat pad. All mice in the control saline‐treatment group had to be euthanized between Days 44 and 48 due to the severity of tumor burdens. On Day 44, all groups treated with DOX and CPT exhibited a statistically significant tumor size reduction relative to the control saline‐treated group irrespective of the dose level (Figure 2a). All combinations were well tolerated based on the effect on body mass (Figure 2b). Dose‐dependent reduction in tumor growth was observed with reductions of 40.8% at 0.5 mg/kg DOX + 0.3 mg/kg CPT to 93% at 1.5 mg/kg DOX + 0.9 mg/kg CPT. The highest tested dose (2 mg/kg DOX + 1.2 mg/kg CPT) completely halted the tumor growth through Day 44.

We continued monitoring the effect of the highest dose (2 mg/kg DOX + 1.2 mg/kg CPT) for an additional 60 days (Figure 2c). We observed that the average progression free survival of the disease was achieved until Day 56. Further, two of the five mice were tumor free survivors whose tumor loads were completely nonapparent by Days 34 and 36 and no cancer recurrence was observed until the study ended. One mouse was a complete responder whose disease progression was prevented until the end of the study. The remaining two mice were partial responders and their tumors grew significantly slower compared to the tumors in the saline treated mice (Figure S3A). Overall, the treatment resulted in a 60% survival rate at the end of a 104‐day study.

### 3.3 Antitumor immune profile after DOX and CPT treatment

We next sought to assess the impact of our drug combination on the tumor immune environment. Since athymic mice lack key adaptive immune system elements, we studied the effect of DOX‐CPT on mouse triple negative 4T1 tumors in immunocompetent balb/c mice. Given the more aggressive nature of 4T1 cells (in vivo doubling time of 4.1 days vs. 11.2 days for MDA‐MB‐231, Figure S5) and the low in vitro synergy of 4T1 cells relative to MDA‐MB‐231 cells, we selected a dose near the highest dose tested in the MDA‐MB‐231 tumor model. Drug doses were 2 mg/kg DOX and 2.5 mg/kg CPT, which correspond to a molar ratio of 1:2 (DOX:CPT), the ratio at which the best in vitro synergy was observed on 4T1 cells (CI = 0.82 ± 0.05).

The DOX‐CPT treated group displayed reduced tumor burden compared to the untreated and single drug treatment groups (Figures S6A and S6B). Also, all treatments were well tolerated as evidenced by the negligible body weight changes (Figure S6C). Although, DOX‐CPT yielded a better therapeutic outcome compared to untreated or individual drugs in terms of tumor growth and survival rates (Figure S6D), the overall efficacy observed for 4T1 was significantly less compared to that for MDA‐MB‐231. The diminished efficacy of DOX‐CPT in the 4T1 breast cancer model could result from either more aggressive 4T1 tumor growth or weak synergistic interactions in 4T1, or both.

It has been previously reported that several chemotherapeutic agents, including DOX, generate antitumor immunogenic effects, but CPT is not believed to not induce such protective immunity.^28^ Moreover, low dose CPT is has been shown to produce an immunosuppressive effect,^29^ and combinations of DOX with other chemotherapy agents have imparted varying levels of both beneficial and detrimental immunity.^30^ We therefore sought to investigate the immune response of the drug combination in the 4T1 syngeneic breast cancer model to determine if any pro‐tumorigenic immune effects were negating the synergistic cytotoxic effect of the drug pair. 4T1 tumors were developed and challenged with the combination and individual drug formulations as described previously, and tumor‐infiltrating immune cells belonging to both innate and adaptive immune response systems were classified (as described in Scheme S1) and subsequently quantified.

**Figure 3:**
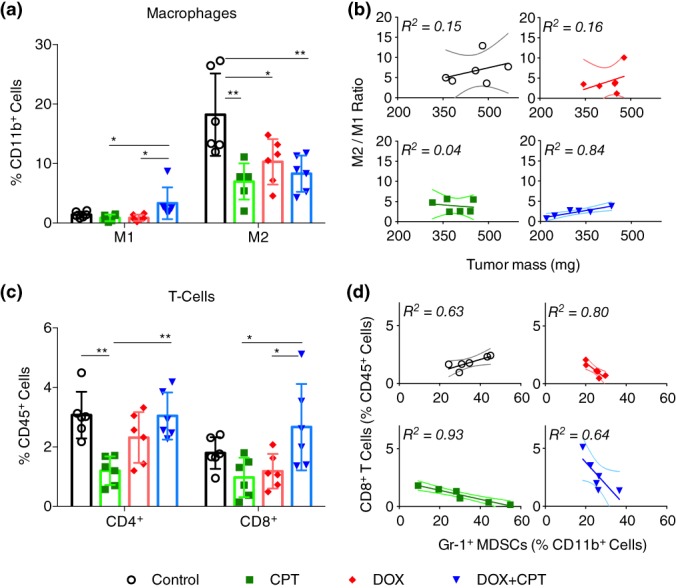
Immune profiling of 4T1 tumors treated with drug cocktails to measure intratumoral levels of tumor‐associated immune cells. Starting on Day 9 postinoculation, mice received four i.v. injections of individual drugs or drug cocktails at drug equivalent doses of 2 mg/kg DOX and/or 2.5 mg/kg CPT. Tumors were excised 8 days after the administration of the last treatment and processed to form single cell suspensions for analysis by flow cytometry. Plots of (a) CD45^+^/CD11b^+^/F4/80^high^/CD80^+^ M1 macrophages, and CD45^+^/CD11b^+^/F4/80^high^/CD206^+^ M2 macrophages, (b) ratio of M2 macrophages to M1 macrophages against tumor mass, (c) CD45^+^/CD3^+^/CD4^+^ T cells and CD45^+^/CD3^+^/CD8^+^ T cells, and (d) CD8^+^ T‐cells against CD45^+^/CD11b^+^/F4/80^low^/gr‐1^+^ myeloid‐derived suppressor cells (MDSCs) for all treatment groups. All data are expressed as mean ± SD (*n* = 5); the solid lines represent the fit obtained from the linear regression analyses performed on the corresponding data and the dotted lines represent their 95% confidence intervals. Statistically significant differences were obtained by performing multiple *t*‐test comparisons. *p < .05 and **p < .01. CPT, camptothecin; DOX, doxorubicin

Tumor associated macrophages (TAMs), one of the most extensively studied cell types of the innate immune system involved in cancer progression, have been previously described to affect the therapeutic outcome of several chemotherapies, including DOX, by mediating the local immunosuppression.^30^ Hence, we began by analyzing the intratumoral levels of CD45^+^/CD11b^+^/F4/80^high^ TAMs. Irrespective of the treatment type, compared to the control untreated group, tumors from all drug treated groups exhibited significantly reduced levels of M2‐like TAMs (CD206^+^) that are associated with pro‐tumor immunosuppressive effects. Secondly, a clear shift and statistically significant in the polarization of TAMs towards the antitumor M1‐phenotype (CD80^+^) was observed for the combination treatment group compared to individual drug treatments (Figure 3a). Within each group, we assessed whether the M2/M1 TAM ratios bore a correlation with the tumor size. For the combination‐treated group, M2/M1 TAM ratio exhibited a significant variation (3.8–0.7) and the ratio exhibited a good correlation with the tumor mass (*R* ^2^ = 0.84). In contrast, only a weak correlation was found for untreated and DOX treated groups, and no correlation was seen in the CPT treated group (Figure 3b). These observations when taken together indicate that a stronger antitumor innate immunity might have been generated in response to the combination treatment compared to the single drug treatments.

We also evaluated the levels of effector T‐cells, which are responsible for mounting an adaptive immune response. Along with disease prognosis, intratumoral levels of CD4^+^ and CD8^+^ T‐cells have been shown to directly impact the efficacy of chemotherapies in several tumor models.^30^ To ensure that we were not counting the immunosuppressive regulatory subset of the CD4^+^ T‐cells, we gated only the CD25^low^/CD4^+^ population. The levels of CD45^+^/CD3^+^/CD4^+^ T‐cells were significantly depleted in the CPT treated group compared to the control group, whereas this difference was insignificant in the DOX treated group. The levels of CD4^+^ T‐cells were restored in mice receiving the combination treatment to those seen in the control group (Figure 3c, left). A similar trend was observed for CD45^+^/CD3^+^/CD8^+^ T‐cells (Figure 3c, right). Although CD8^+^ T‐cells were present in significantly higher amounts in tumors that received the combination treatment compared to those that received single drug treatments, the levels were not significantly higher than those observed within the control group.

We next plotted the number of CD8^+^ T‐cells against CD45^+^/CD11b^+^/F4/80^low^/Gr1^+^ myeloid‐derived suppressor cells (MDSCs), which can foster chemoresistance by depleting cytotoxic cells.^30^ As expected, a strong negative correlation was observed for all treated groups (i.e., higher CD8^+^ T‐cell counts were observed in the absence of Gr1^+^ MDSCs; Figure 3d). However, we noted that the absolute levels of Gr1^+^ MDSCs were comparable in all groups (Figure S7A). In contrast to the negative correlation seen in the treated groups, a positive correlation between CD8+ T cells and Gr1^+^ MDSCs was observed in the control group. This surprising trend could indicate a possible dampening of the adaptive response due to higher MDSCs, despite having higher absolute numbers of CD8^+^ T‐cells.^31^ Interestingly, the numbers of dendritic cells were significantly higher in groups receiving DOX treatments, either individually or in combination with CPT, when compared to the CPT treatment group (Figure S7B). However, the differences between the control and DOX treated groups or between the two DOX treatment groups were insignificant.

## 4 DISCUSSION

Identifying clinically successful synergistic combinations is a challenging task and is often based on selecting drugs that exhibit uncorrelated inhibitory effects and orthogonal toxicities.^32^ DOX and CPT are an excellent choice for combination from this point of view since they operate via cross‐sensitive drug interactions.^14^, ^22^, ^23^ CPT and DOX are topoisomerase I and II inhibitors respectively, and in combination exhibit collateral drug sensitivity or in other words sensitize cancer cells to one‐another and synergistically hinder tumor growth.^33^ Furthermore, they have distinct dose‐limiting toxicities. While doxorubicin's main adverse effect is cardiomyopathy, camptothecin primarily induces myelosuppression.^21^, ^34^

In agreement with previous reports, we noticed a ratio‐dependent synergy between DOX and CPT for several breast cancer cell lines in vitro (Figure 1). Further investigation revealed that regardless of the difference in the relative susceptibility of cells to DOX and CPT, compositions with relatively greater amounts of CPT conferred higher synergies in all cell types. While detailed mechanistic studies are needed to explain such behavior, one possible explanation can be derived from previous findings, which have reported that treating cells with DOX prior to CPT led to antagonism but concomitant or reverse schedules are not antagonistic.^35^ Ratio‐dependent synergy could be a manifestation of such schedule‐dependent effects^36^ (i.e., ratios with higher relative amounts of DOX may reflect exposure of DOX prior to CPT), thus leading to antagonism. Secondly, stronger synergy was exhibited in cell lines that were more susceptible to CPT compared to DOX. Taken together, the results imply that higher synergies are obtained when CPT affects a larger fraction of the tumor cell population, either because the combination consist a larger proportion of CPT or due to its superior relative efficacy.

DOX and CPT combined at an equimolar ratio were highly synergistic in MDA‐MB‐231 cells in vitro. The DOX dose was reduced by 86% (seven‐fold) and the CPT dose was reduced by 68% (three‐fold) in these cells. If the drugs had a purely additive effect, the dose reductions would be inversely proportional to the ratios in which they were combined. A 1:1 molar ratio of DOX:CPT should have resulted only in a 50% (two‐fold) dose reduction for each drug. Additionally, this synergistic response was carried forward in vivo, where cumulative doses as low as 1 mg/kg DOX + 0.6 mg/kg CPT and 2 mg/kg DOX + 1.2 mg/kg CPT were able to induce measurable and significant tumor shrinkages. In fact, the tumor growth inhibitions obtained at these doses were far better than those gained at a cumulative dose of 8 mg/kg DOX^37^ or comparable to the tumor growth inhibition previously obtained at a cumulative dose of 30 mg/kg CPT.^38^ Remarkably, our highest dose level (2 mg/kg DOX + 1.2 mg/kg CPT) achieved striking therapeutic efficacies in vivo, with 60% of the mice having either completely eradicated tumors or progression free tumor burdens until the end of the study (Figure 2). This dose corresponds to a twofold reduction in the dosage of DOX (cumulative dose of 8 mg/kg), when compared to a previous study that reported similar tumor regression on MDA‐MB‐231 tumors treated with a cumulative DOX dose of 16 mg/kg.^39^ The reduction in the CPT dose was even larger. A previous in vivo study reported 49% tumor suppression after administering 30 mg/kg CPT.^38^ Compared to this, a significant reduction in tumor burden was obtained at a six‐fold lower dose of CPT. It is worthwhile to note that these in vivo dose reduction levels are comparable in magnitude to our in vitro reduction levels. Subsequent studies done in the syngeneic 4T1 breast cancer model also showed that the dual drug combination was significantly better at inhibiting tumor growth compared to the individual drug treatments, which provided little to no benefit in preventing disease progression (Figure S6). Overall, these results strongly indicate that the biochemical synergistic interactions observed between DOX and CPT in vitro are most likely retained in vivo.

One of the main concerns of chemotherapeutic combinations, even if deemed synergistic in preclinical studies, is that they have very poor therapeutic indices due to the compounding of their individual toxicities.^29^ Nevertheless, in our dose titration studies, we saw that minor dose increments (0.5 mg/kg DOX + 0.3 mg/kg CPT) led to drastic improvements in efficacy without corresponding affecting the tolerability. Crucially, the potent response in managing the MDA‐MB‐231 tumor burden was achieved at doses of DOX and CPT that were roughly five‐fold and ten‐fold lower than their reported MTDs, respectively (DOX (between 8 and 12 mg/kg/dose)^25^, ^26^ and CPT (15 mg/kg/dose)^27^).

Regardless of the effective responses observed in the MDA‐MB‐231 model, the drug pair was less successful in mitigating the burden of 4T1 tumors. At the dose used in this study, the combination inhibited 4T1 tumor growth less effectively than the inhibition obtained at an eight‐fold lower dose in MDA‐MB‐231 tumors, even if the 4T1 tumor volumes doubled only 2–3 times as aggressively as the MDA‐MB‐231 tumor volumes. This disparity could either be a result of the difference in synergies observed between the two cell lines in vitro or due to the differences in the immune systems of the host mice.

Immunogenic chemotherapy combinations administered at sublethal doses have led to mixed antitumor immune outcomes. For example, a combination of doxorubicin and lapatinib resulted in improved antitumor responses by enhancing the infiltration of IFN‐γ secreting CD4^+^ and CD8^+^ T cells into the mammary tumors of HER2^+^ breast cancer bearing mice.^40^ However, when vincristine was added to a metronomic cyclophosphamide‐doxorubicin combination, previously shown to induce antitumor adaptive immunity,^41^ the T cell and natural killer cell growth was muted in a B16 melanoma model.^42^ Our studies of the local tumor immune microenvironment (Figure 3) revealed that DOX‐CPT in combination drove the polarization of TAMs towards an antitumor M1‐like phenotype in lieu of the pro‐tumor M2‐like phenotype. The single drug treatments also displayed reduced pro‐tumor M2‐like TAMs compared to the control group; however, a strong correlation in the M2/M1 TAM ratio with tumor size was evident only in the dual drug treated group. The weaker correlation in the DOX treated group and the lack of correlation in the CPT treated group suggests that a stronger antitumor innate immune response is present in the dual drug treated tumors. Prior studies have suggested that DOX, administered either alone or in a combination with other drugs, is capable of enhancing the tumoricidal properties of TAMs.^8^, ^43^ Other than the low antitumor immune activation of DOX, which could be a result of an insufficient concentration of DOX at the tumor site, our results are mostly consistent with these findings. Reports from other studies have proposed that eliminating Gr1^+^ MDSCs can augment the latent tumor immunity by preventing the exhaustion of cytotoxic T‐cells.^31^ Whereas an opposite trend was observed in the control group, similar trends were observed in all drug treated groups. Further, in agreement with previous findings, we also observed a significant reduction of CD4^+^ and CD8^+^ T‐cells in CPT treated tumors, but these levels were restored to original levels in the combination treatment.^44^ Lastly, the levels of dendritic cells were significantly higher in groups receiving DOX alone or the drug combination when compared to those receiving CPT alone (Figure S6B), which indicates a mounting adaptive response. This behavior is expected since DOX has been previously shown to provide protective antitumor immunity by enhancing the recruitment and maturation of dendritic cells.^45^ Cumulatively, these results suggest that a favorable antitumor immune response is established by the drug combination.

While DOX is routinely employed in the clinic, CPT has been less successful in its translation perhaps in part due to its poor water solubility.^46^ Initial efforts to increase its solubility at neutral pH were mostly focused on opening of its lactone ring, which resulted in a dramatic reduction of its cytotoxicity. As a countermeasure, very high doses of CPT were administered to achieve meaningful efficacies, which resulted in severe myelosuppression.^21^ In addition to the unpredictable toxicity in patients, variable and limited objective responses in phase II clinical trials led to its failure.^21^ To overcome problems associated with CPT, water soluble derivatives like irinotecan and topotecan were discovered and used along with DOX.^47^, ^48^ Their success is however limited due to significant worsening in toxicity and minimal therapeutic benefit after combining them. Further, the decreased antitumor activity compared to their water insoluble counterparts,^21^, ^46^ instigated a wide effort to find alternative ways to translate them.

Parallel efforts to revive CPT have shown that covalent conjugation at the 20‐OH position to water soluble polymers can stabilize the labile lactone ring and improve solubility.^49^, ^50^ Several vectors for delivering CPT have been developed and are actively undergoing clinical investigation.^49^, ^50^ These technologies were developed with a focus on improving the solubility, pharmacokinetic properties and reducing the adverse reactions of CPT to enhance its therapeutic window, tolerability and tumor accumulation levels. Although moderate antitumor activities were observed for several of these drug conjugates, clinical advancement is stunted due to the bladder toxicity from high levels of camptothecin excreted via urine.^51^ Studies presented here show that CPT when combined with DOX can produce synergy that can translate into effective tumor reductions at highly reduced drug doses. This alternative strategy could counter the severe toxicity issues that arise as part of the MTD approach. Ultimately, potent drugs like CPT that have failed in the clinic due to high dose administrations can be reintroduced. However, moving forward, investigation on the overlapping toxicities of DOX and CPT, such as kidney toxicity, gastrointestinal toxicities and dermatitis, is needed. Although these toxicities are not dose‐limiting when DOX and CPT are administered separately, studies evaluating their risk after concurrent administration at higher doses are necessary.^34^, ^46^

Currently, ratio‐dependent behaviors are difficult to predict without empirical testing. Future studies focused on understanding drug interactions in mechanistic detail can help in developing more rational methods to choose such combinations. Furthermore, formulations that can unify the pharmacokinetics and co‐deliver DOX and CPT in specific molar ratios to the tumor tissue can be developed for improving the therapeutic efficacy even further. A few nano‐formulations are being developed for such a therapy, including hyaluronic acid drug conjugates, polymeric nanoparticles and targeted delivery systems.^14^, ^22^, ^52^ Further myelosuppression, the most common side effect of high dose chemotherapies, has so far limited the usage of chemotherapy as an effective adjuvant or neoadjuvant therapy in combination with immunotherapies.^6^ Chemotherapy combinations like the one studies here, which produce sufficient cytotoxicity and immunostimulatory effects at low doses and, should be further exploited for different multi‐platform combination therapies.^31^

## 5 CONCLUSION

Severe toxicity issues have impaired the clinical progress of combination chemotherapy, but several preclinical studies and clinical studies outlining ways to mitigate this toxicity foreshadow its reemergence. By controlling drug molar ratios and schedules of chemotherapeutics, higher efficacies at low doses have been obtained.^11^, ^53^, ^54^ We propose an optimized combination of DOX and CPT as a low dose therapy option for aggressive breast cancer. DOX and CPT were found to be a potent drug pair that exhibited molar ratio‐dependent synergy against human triple negative breast cancer cells, MDA‐MB‐231. By optimizing molar ratios of the drug pair through systematic screening, high efficacies at extremely low doses, roughly five‐fold lower than the MTD of individual drugs, were obtained in an in vivo orthotopic MDA‐MB‐231 murine model. The doses used were very well tolerated but the drug pair was unable to produce such high efficacies in a 4T1 murine model, possibly due to the low in vitro synergy of the combination observed on this cell line. However, they were able to induce a favorable anticancer immune response, which could potentially interact with both passive and active immunotherapy strategies in a synergistic fashion and inhibit tumor growth in more challenging scenarios.^8^ Drug combinations can be extremely beneficial, but careful engineering is necessary for their effective translation into the clinic. In summary, this body of work demonstrates the feasibility of designing viable low dose therapeutic option for combination cancer therapy by careful molar ratio optimization.

## Supporting information


**Table S1** Antibodies used in the experiment
**Table S2:** Statistical Analyses for Figure 1.
**Table S3:** Statistical Analyses for Figure 3D.
**Figure S1**. Individual toxicities (IC_50_) of DOX and CPT on MDA‐MB‐231, MCF 7 and 4T1 cell lines. Relative sensitivity between DOX and CPT is defined as the ratio of DOX IC_50_ to CPT IC_50_. Data are expressed as mean ± standard error obtained from the drug model fits (n ≥ 5).
**Figure S2**. Effect of molar ratios on the proliferation (left) and synergy (right) in (A) MDA‐MB‐231, (B) MCF 7 and (C) 4T1 cells. IC_50_ values are expressed as mean ± standard error obtained from the drug model fits (n ≥ 5) and combination indices were calculated at various effect levels using the Chou‐Talalay method. The black line represents CI = 1 (additive effect), CI < 1 is synergistic and CI > 1 is antagonistic. All statistical analyses are provided in the supplementary information in Table S2.
**Figure S3**. (A) Individual tumor growth curves of mice treated with DOX and CPT at 2 mg/kg DOX and 1.2 mg/kg CPT. # ‐ indicates when the mice were euthanized. Tumors from mouse 2 and mouse 5 were no longer palpable from Day 36 and 34, respectively. (B) Body weight changes in tumor‐bearing mice that received either a saline treatment or a treatment of DOX and CPT at a 1:1 MM ratio. Data are expressed as mean ± SEM (n = 5). (C) Kaplan–Meier survival curve following i.v. administration of saline or DOX + CPT cocktail at 2 mg/kg DOX and 1.2 mg/kg CPT in athymic nu/nu mice carrying MDA‐MB‐231 orthotopic breast tumors. Starting on Day 11 post‐inoculation, four i.v. injections were administered every other day. Mice were observed for 104 days and were euthanized if tumor length exceeded 15 mm, or if body weight loss was greater than 15%.
**Figure S4**. Tumor growth inhibitions of orthotopic MDA‐MB‐231 mouse breast tumors, calculated on Day 44, after combination treatments with DOX and CPT at different dose levels. Starting on the Day 11 post‐inoculation, four i.v. injections were administered every other day. Statistically significant differences are shown based on the multiple T‐test comparisons performed on the last day on the growth in tumor volume curve (Day 44). Data are expressed as mean ± SEM (n = 5). *p < 0.1, **p < 0.01, ****p < 0.0001.
**Figure S5**. Tumor growth curves for (A) balb/c mice bearing 4T1 orthotopic breast tumors and (B) athymic nu/nu mice bearing MDA‐MB‐231 orthotopic breast tumors that did not receive any treatment. Data are expressed as mean ± SEM (n = 5). Dotted lines represent exponential fits to tumor doubling times. 4T1 tumor doubling time = 4.1 days and MDA‐MB‐231 tumor doubling time = 11.2 days.
**Figure S6**. DOX and CPT in vivo efficacy in an orthotopic 4T1 mouse breast cancer model. (A) Tumor growth curves for untreated or DOX and CPT treated mice that received four i.v. injections starting on the Day 9 post‐inoculation. Drugs were administered either individually or in combination at a molar ratio of 1:2 at drug equivalent doses of 2 mg/kg DOX and 2.5 mg/kg CPT. Statistically significant differences are shown for the last day on the growth curve (Day 23). *p < 0.05, **p < 0.01, ***p < 0.001 and ****p < 0.0001. (B) Corresponding weights of tumors excised on Day 23 and (C) body weight changes for all treatment groups. (D) Kaplan–Meier survival for untreated or DOX and CPT treated balb/c mice carrying 4T1 orthotopic breast tumors that received treatments as described above. Mice were euthanized if tumor length exceeded 15 mm, or if body weight loss was greater than 15%. Statistically significant differences have been listed in Table S3. All data are expressed as mean ± SEM (n = 5).
**Figure S7**. Immune cell profiling of 4T1 tumors treated with drug cocktails. Starting on Day 9 post‐inoculation, mice received four i.v. injections of individual drugs or drug cocktails at drug equivalent doses of 2 mg/kg DOX and/or 2.5 mg/kg CPT. Tumors were excised 8 days after the administration of the last treatment and processed to form single cell suspensions for analysis by flow cytometry. Plots of (A) CD45^+^/CD11b^+^/F4/80^low^/Gr‐1^+^ myeloid‐derived suppressor cells (MDSCs) and (B) CD45^+^/CD11b^+^/F4/80^low^/CD11c^+^ dendritic cells for all treatment groups. All data are expressed as mean ± SD (n = 5). Statistically significant differences were obtained by performing multiple T‐test comparisons. *p < 0.05, and ***p < 0.001.
**Scheme S1**. Strategy for phenotyping tumor‐associated immune cells in orthotopic 4T1 breast carcinoma. Live cells underwent 3 separate treatments, as demarcated by the dotted gray boxes. Phenotypic markers used for identifying different cell types are shown in red text. Cell populations shown in green boxes are known to be responsible for producing anti‐tumor immunogenic responses, while populations shown in magenta boxes are known to elicit pro‐tumor immunosuppressive effects.
