# Supplementary material for: Role of synergy and immunostimulation in design of chemotherapy combinations: An analysis of doxorubicin and camptothecin
Source: Bioeng Transl Med. 2019 Jun 13;4(2):e10129. doi: 10.1002/btm2.10129 (PMC6584462; doi:10.1002/btm2.10129)
Supplement: Supplementary file 1 — Table S1 Antibodies used in the experiment Table S2: Statistical Analyses for Figure 1. Table S3: Statistical Analyses for Figure 3D. Figure S1. Individual toxicities (IC50) of DOX and CPT on MDA‐MB‐231, MCF 7 and 4T1 cell lines. Relative sensitivity between DOX and CPT is defined as the ratio of DOX IC50 to CPT IC50. Data are expressed as mean ± standard error obtained from the drug model fits (n ≥ 5). Figure S2. Effect of molar ratios on the proliferation (left) and synergy (right) in (A) MDA‐MB‐231, (B) MCF 7 and (C) 4T1 cells. IC50 values are expressed as mean ± standard error obtained from the drug model fits (n ≥ 5) and combination indices were calculated at various effect levels using the Chou‐Talalay method. The black line represents CI = 1 (additive effect), CI < 1 is synergistic and CI > 1 is antagonistic. All statistical analyses are provided in the supplementary information in Table S2. Figure S3. (A) Individual tumor growth curves of mice treated with DOX and CPT at 2 mg/kg DOX and 1.2 mg/kg CPT. # ‐ indicates when the mice were euthanized. Tumors from mouse 2 and mouse 5 were no longer palpable from Day 36 and 34, respectively. (B) Body weight changes in tumor‐bearing mice that received either a saline treatment or a treatment of DOX and CPT at a 1:1 MM ratio. Data are expressed as mean ± SEM (n = 5). (C) Kaplan–Meier survival curve following i.v. administration of saline or DOX + CPT cocktail at 2 mg/kg DOX and 1.2 mg/kg CPT in athymic nu/nu mice carrying MDA‐MB‐231 orthotopic breast tumors. Starting on Day 11 post‐inoculation, four i.v. injections were administered every other day. Mice were observed for 104 days and were euthanized if tumor length exceeded 15 mm, or if body weight loss was greater than 15%. Figure S4. Tumor growth inhibitions of orthotopic MDA‐MB‐231 mouse breast tumors, calculated on Day 44, after combination treatments with DOX and CPT at different dose levels. Starting on the Day 11 post‐inoculation, four i.v. injections were adm [file BTM2-4-na-s001.docx]

**Supplementary material**

**Table S1:** Antibodies used in the experiment

| Antibody target (fluorophore) | Host / Isotype | Clone | Supplier |
| --- | --- | --- | --- |
| F4/80/ (PerCP-Cy5.5) | Rat / IgG2a, kappa | BM8.1 | MilliporeSigma |
| CD45 (FITC) | Rat / IgG2b, kappa | 30-F11 | ThermoFisher Scientific |
| CD11b (APC) | Rat / IgG2b, kappa | M1/70 |  |
| CD3e (PerCP-Cy5.5) | Armenian hamster / IgG | 145-2C11 |  |
| CD25 (APC) | Rat / IgG1, lambda | PC61.5 |  |
| CD8a (PE) | Rat / IgG2a, kappa | 53-6.7 |  |
| CD4 (PE-Cy7) | Rat / IgG2a, kappa | RM4-5 |  |
| Ly-6G/Ly-6C (PE-Cy7) | Rat / IgG2b, kappa | RB6-8C5 |  |
| CD11c (PE) | Armenian hamster / IgG | N418 |  |
| CD80 (PE) | Armenian hamster / IgG | 16-10A1 |  |
| CD206 (PE-Cy7) | Rat / IgG2b, kappa | MR6F3 |  |
| Isotype Control (FITC) | Rat / IgG2b, kappa | eB149/10H5 |  |
| Isotype Control (APC) | Rat / IgG2b, kappa | eB149/10H5 |  |
| Isotype Control (PerCP-Cy5.5) | Armenian hamster / IgG | eBio299Arm |  |
| Isotype Control (APC) | Rat / IgG1, kappa | eBRG1 |  |
| Isotype Control (PE-Cy7) | Rat / IgG2a, kappa | eBR2a |  |
| Isotype Control (PE) | Rat / IgG2a, kappa | eBR2a |  |
| Isotype Control (PerCP-Cy5.5) | Rat / IgG2a, kappa | eBR2a |  |
| Isotype Control (PE-Cy7) | Rat / IgG2b, kappa | eB149/10H5 |  |
| Isotype Control (PE) | Armenian hamster / IgG | eBio299Arm |  |

**Table S2:** Statistical Analyses for Fig 1.

| Ratio | IC_50_ DOX | | | IC_50_ CPT | | |
| --- | --- | --- | --- | --- | --- | --- |
|  | MDA-MB-231 | MCF 7 | 4T1 | MDA-MB-231 | MCF 7 | 4T1 |
| 4:1 vs. 3:1 | ** | **** | **** | ns | * | ns |
| 4:1 vs. 2:1 | **** | **** | ns | ns | ** | **** |
| 4:1 vs. 1:1 | **** | **** | **** | ns | ns | **** |
| 4:1 vs. 1:2 | **** | **** | **** | ns | ns | **** |
| 4:1 vs. 1:3 | **** | **** | **** | ns | **** | **** |
| 4:1 vs. 1:4 | **** | **** | **** | ns | **** | **** |
| 3:1 vs. 2:1 | **** | **** | **** | * | ns | **** |
| 3:1 vs. 1:1 | **** | * | **** | ** | **** | **** |
| 3:1 vs. 1:2 | **** | **** | **** | ns | **** | **** |
| 3:1 vs. 1:3 | **** | **** | **** | ns | **** | **** |
| 3:1 vs. 1:4 | **** | **** | **** | ns | **** | **** |
| 2:1 vs. 1:1 | **** | ns | **** | ns | **** | **** |
| 2:1 vs. 1:2 | **** | * | **** | ns | **** | **** |
| 2:1 vs. 1:3 | **** | ns | **** | * | **** | **** |
| 2:1 vs. 1:4 | **** | * | **** | ns | **** | **** |
| 1:1 vs. 1:2 | ns | *** | **** | ns | ns | **** |
| 1:1 vs. 1:3 | ns | *** | **** | ** | **** | **** |
| 1:1 vs. 1:4 | * | *** | **** | ns | **** | **** |
| 1:2 vs. 1:3 | ns | ns | ns | ns | **** | **** |
| 1:2 vs. 1:4 | ns | ns | **** | ns | **** | **** |
| 1:3 vs. 1:4 | ns | ns | *** | ns | * | ** |

| Ratio | Chou-Talalay Combination Index (CI) differences between different cell lines | | |
| --- | --- | --- | --- |
|  | MDA-MB-231 vs. 4T1 | MDA-MB-231 vs. MCF7 | MCF7 vs. 4T1 |
| 4:1 | ns | **** | **** |
| 3:1 | ns | **** | **** |
| 2:1 | **** | ns | **** |
| 1:1 | **** | **** | * |
| 1:2 | *** | ns | ns |
| 1:3 | ** | **** | ns |
| 1:4 | *** | **** | **** |

| Ratio | CI differences within same cell line | | |
| --- | --- | --- | --- |
|  | MDA-MB-231 | MCF 7 | 4T1 |
| 4:1 vs. 3:1 | ns | **** | ns |
| 4:1 vs. 2:1 | **** | **** | ns |
| 4:1 vs. 1:1 | **** | **** | *** |
| 4:1 vs. 1:2 | **** | **** | **** |
| 4:1 vs. 1:3 | **** | **** | **** |
| 4:1 vs. 1:4 | **** | **** | **** |
| 3:1 vs. 2:1 | **** | *** | *** |
| 3:1 vs. 1:1 | **** | ns | ns |
| 3:1 vs. 1:2 | **** | ns | **** |
| 3:1 vs. 1:3 | **** | ns | *** |
| 3:1 vs. 1:4 | **** | *** | **** |
| 2:1 vs. 1:1 | ns | **** | **** |
| 2:1 vs. 1:2 | ns | ns | **** |
| 2:1 vs. 1:3 | ns | **** | **** |
| 2:1 vs. 1:4 | ns | **** | **** |
| 1:1 vs. 1:2 | ns | ** | ** |
| 1:1 vs. 1:3 | ns | ns | ns |
| 1:1 vs. 1:4 | ns | * | ** |
| 1:2 vs. 1:3 | ns | **** | ns |
| 1:2 vs. 1:4 | ns | **** | ns |
| 1:3 vs. 1:4 | ns | ns | ns |

* = p < 0.05, ** = p < 0.01, *** = p < 0.001, **** = p < 0.0001, ns = not significant

**Table S3:** Statistical Analyses for Fig 3D.

|  | Untreated | DOX | CPT | DOX + CPT |
| --- | --- | --- | --- | --- |
| Untreated | - | ** | ns | ** |
| DOX |  | - | * | * |
| CPT |  |  | - | ** |
| DOX + CPT |  |  |  | - |

* = p < 0.05, ** = p < 0.01, ns = not significant

**Fig S1. Individual toxicities (IC_50_) of DOX and CPT on MDA-MB-231, MCF 7 and 4T1 cell lines. Relative sensitivity between DOX and CPT is defined as the ratio of DOX IC_50_ to CPT IC_50_. Data are expressed as mean ± standard error obtained from the drug model fits (n ≥ 5).**

**Fig S2. Effect of molar ratios on the proliferation (left) and synergy (right) in (A) MDA-MB-231, (B) MCF 7 and (C) 4T1 cells. IC_50_ values are expressed as mean ± standard error obtained from the drug model fits (n ≥ 5) and combination indices were calculated at various effect levels using the Chou-Talalay method. The black line represents CI = 1 (additive effect), CI < 1 is synergistic and CI > 1 is antagonistic. All statistical analyses are provided in the supplementary information in Table S2.**

**Fig S3. (A) Individual tumor growth curves of mice treated with DOX and CPT at 2 mg/kg DOX and 1.2 mg/kg CPT. # - indicates when the mice were euthanized. Tumors from mouse 2 and mouse 5 were no longer palpable from Day 36 and 34, respectively. (B) Body weight changes in tumor-bearing mice that received either a saline treatment or a treatment of DOX and CPT at a 1:1 molar ratio. Data are expressed as mean ± SEM (n = 5). (C) Kaplan-Meier survival curve following i.v. administration of saline or DOX+CPT cocktail at 2 mg/kg DOX and 1.2 mg/kg CPT in athymic nu/nu mice carrying MDA-MB-231 orthotopic breast tumors. Starting on Day 11 post-inoculation, four i.v. injections were administered every other day. Mice were observed for 104 days and were euthanized if tumor length exceeded 15 mm, or if body weight loss was greater than 15%.**

**Fig S4. Tumor growth inhibitions of orthotopic MDA-MB-231 mouse breast tumors, calculated on Day 44, after combination treatments with DOX and CPT at different dose levels. Starting on the Day 11 post-inoculation, four i.v. injections were administered every other day. Statistically significant differences are shown based on the multiple T-test comparisons performed on the last day on the growth in tumor volume curve (Day 44). Data are expressed as mean ± SEM (n = 5). * = p < 0.1, ** = p < 0.01, **** = p < 0.0001.**

**Fig S5. Tumor growth curves for (A) balb/c mice bearing 4T1 orthotopic breast tumors and (B) athymic nu/nu mice bearing MDA-MB-231 orthotopic breast tumors that did not receive any treatment. Data are expressed as mean ± SEM (n = 5). Dotted lines represent exponential fits to tumor doubling times. 4T1 tumor doubling time = 4.1 days and MDA-MB-231 tumor doubling time = 11.2 days.**

**Fig S6. DOX and CPT *in vivo* efficacy in an orthotopic 4T1 mouse breast cancer model. (A) Tumor growth curves for untreated or DOX and CPT treated mice that received four i.v. injections starting on the Day 9 post-inoculation. Drugs were administered either individually or in combination at a molar ratio of 1:2 at drug equivalent doses of 2 mg/kg DOX and 2.5 mg/kg CPT. Statistically significant differences are shown for the last day on the growth curve (Day 23). * = p < 0.05, ** = p < 0.01, *** = p < 0.001 and **** = p < 0.0001. (B) Corresponding weights of tumors excised on day 23 and (C) body weight changes for all treatment groups. (D) Kaplan-Meier survival for untreated or DOX and CPT treated balb/c mice carrying 4T1 orthotopic breast tumors that received treatments as described above. Mice were euthanized if tumor length exceeded 15 mm, or if body weight loss was greater than 15%. Statistically significant differences have been listed in Table S3. All data are expressed as mean ± SEM (n = 5).**

**Fig S7. Immune cell profiling of 4T1 tumors treated with drug cocktails. Starting on Day 9 post-inoculation, mice received four i.v. injections of individual drugs or drug cocktails at drug equivalent doses of 2 mg/kg DOX and/or 2.5 mg/kg CPT. Tumors were excised 8 days after the administration of the last treatment and processed to form single cell suspensions for analysis by flow cytometry. Plots of (A) CD45^+^/CD11b^+^/F4/80^low^/Gr-1^+^ myeloid-derived suppressor cells (MDSCs) and (B) CD45^+^/CD11b^+^/F4/80^low^/CD11c^+^ dendritic cells for all treatment groups. All data are expressed as mean ± SD (n = 5). Statistically significant differences were obtained by performing multiple T-test comparisons. * = p < 0.05, and *** = p < 0.001.**

**
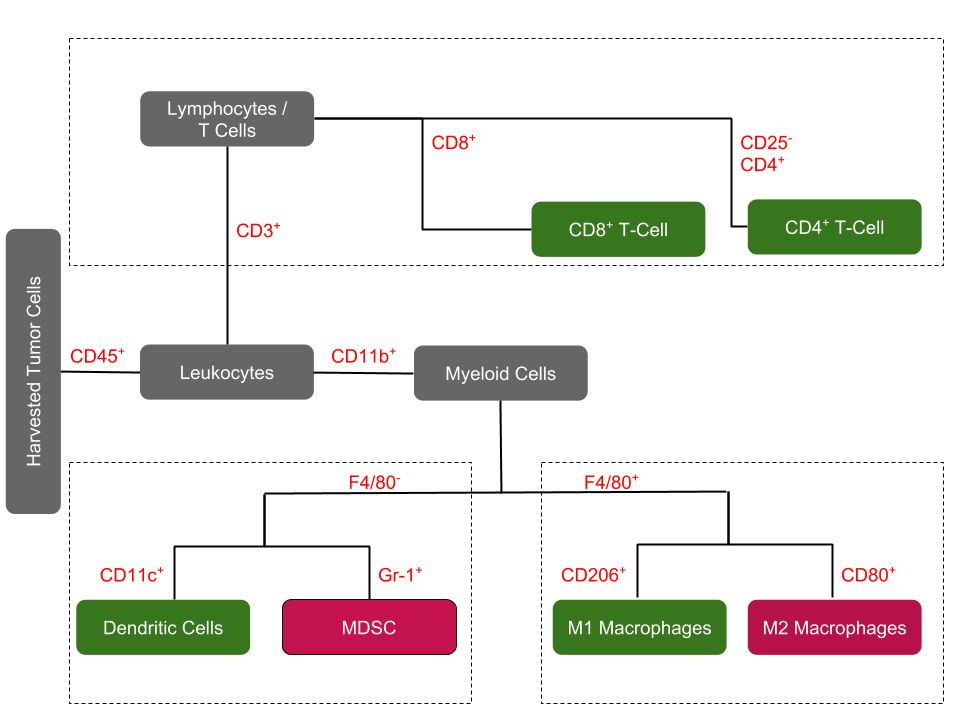
**

**Scheme S1. Strategy for phenotyping tumor-associated immune cells in orthotopic 4T1 breast carcinoma. Live cells underwent 3 separate treatments, as demarcated by the dotted grey boxes. Phenotypic markers used for identifying different cell types are shown in red text. Cell populations shown in green boxes are known to be responsible for producing anti-tumor immunogenic responses, while populations shown in magenta boxes are known to elicit pro-tumor immunosuppressive effects.**
